# Yeasts Associated with the Small-Intestinal Contents and Epithelium of Pon Yang Kham (Charolais Crossbred) Fattening Beef Cattle

**DOI:** 10.3390/microorganisms9071444

**Published:** 2021-07-04

**Authors:** Jirameth Angchuan, Pannida Khunnamwong, Kannika Wongpanit, Savitree Limtong, Nantana Srisuk

**Affiliations:** 1 Department of Microbiology, Faculty of Science, Kasetsart University, Bangkok 10900, Thailand; jirameth.a@ku.th (J.A.); pannida.k@ku.th (P.K.); fscistl@ku.ac.th (S.L.); 2 Biodiversity Center, Kasetsart University (BDCKU), Bangkok 10900, Thailand; 3 Faculty of Natural Resources and Agro-Industry, Chalermphrakiat Sakon Nakhon Province Campus, Kasetsart University, Sakon Nakhon 47000, Thailand; kannika_ku@yahoo.com; 4 Academy of Science, The Royal Society of Thailand, Bangkok 10300, Thailand

**Keywords:** yeast, pia, small-intestinal epithelium, cattle

## Abstract

Yeast diversity in the pia and small-intestinal epithelium of Pon Yang Kham fattening cattle in Thailand was studied using a culture-dependent method. A total of 701 yeasts were isolated from the pia of the duodenum, jejunum, and ileum of the small intestine, while 425 isolates were obtained from the epithelium of all three parts of the small intestine. Yeast identification was performed and ascomycetous yeasts were found at levels of 96.9% and 86.8% in the pia and small intestine, respectively, whereas basidiomycetous yeasts were found at levels of 2.3% and 12.7%. *Candida parapsilosis* was the species with the highest occurrence in the duodenal and jejunal pia, with an 83.3% and 77.8% frequency of occurrence (FO), respectively. Both *C. parapsilosis* and *C. tropicalis* were species with the highest occurrence in the ileum, with a 61.1% FO. Moreover, *C. parapsilosis* was the species with the highest occurrence in the epithelium of the duodenum, jejunum, and ileum, with FOs of 88.2%, 87.5%, and 87.2%, respectively. Principal coordinate analysis revealed no marked differences in yeast communities from either the pia or epithelium of all three parts of the small intestine. An estimation of the expected richness of the species showed that the observed species richness was lower than the predicted richness.

## 1. Introduction

Pon Yang Kam fattening beef cattle are Charolais crossbred cattle and have improved the quality of beef in response to consumer demand by the Pon Yang Kham Livestock cooperatives in Sakon Nakhon Province, Thailand. These cattle were fed natural feed and crude protein comprised of casava, rice bran, palm kernel meal, molasses and urea, which were provided by the cooperative. In addition, they were not treated with hormones, antibiotics, imported feed ingredients or artificial vitamins [1]. Moreover, based on data from 2013 to 2017, the number of Pon Yang Kham fattening cattle is increasing [2]. In Thailand, the fluid inside the small intestine of cattle is well known to be pia. In the past, in animal husbandry in Thailand, pia has been a dietary component used to treat sick beef cattle. Currently, pia is popularly used as a cooking ingredient in many traditional dishes from Northeast Thailand because of its unique taste.

The animal gastrointestinal tract is a complex ecosystem of microorganisms, including bacteria, archaea, fungi, and protozoa [3]. Moreover, a large number of microorganisms, known as the microbiota, are also found on the epithelial surface of the mammalian intestine [4]. Infection leads to dysbiosis in both humans and animals [5,6]. However, many studies have reported several cases of human diseases, including inflammatory bowel disease (IBD) [7], metabolic diseases, such as obesity and diabetes [8] and allergy-related diseases [9], which were caused by an imbalance in gut microorganisms. However, microbes are also functional in several metabolic pathways and provide benefits to the host—for example, in strengthening the gut barrier function, shaping intestinal epithelial cells [10], regulating and modulating host immunity [11], degrading complex substances for the host [12,13], and defending against pathogens [14].

Yeasts have been discovered in many ecosystems and play an important role in the biodiversity of Earth [15]. Several studies on yeast diversity in the digestive tract of animal species have been conducted—for example, studies on the occurrence and diversity of yeast in fish feed and the tambatinga gut [16], lactic acid-utilizing yeasts from dairy cattle ruminal fluid [17], potential probiotic yeasts from the bovine rumen [18] and a novel yeast species, *Methanobrevibacter boviskoreani*, from the Korean native cattle rumen [19]. However, the microorganisms in pia have not yet been assessed. Furthermore, assessment of the yeast community in the animal gut is important and yields interesting results; for example, the yeast flora in the ceca of healthy bovines, horses, sheep, goats, and swine has been characterized, and the proportions of samples containing yeasts were found to be 46.8%, 52.4%, 6.8%, 6.4% and 88.8%, respectively. *Candida tropicalis*, *Candida sloofii*, *Trichosporon cutaneum*, *Pichia kudriavzevii* and *Candida albicans* were reported as the most frequently occurring yeasts in the ceca of bovines, swine, horses, goats and sheep, respectively [20,21]. In addition, the prevalence of yeast in the gastrointestinal tract of turkeys was investigated. The most frequently found yeast genera were *Candida* (88.5%), *Trichosporon* (7.7%), and *Rhodotorula* (3.5%) [22]. While some yeast species, such as *C. albicans* and *C. tropicalis*, were reported as opportunistic pathogens in animals [23], some species, such as *Saccharomyces cerevisiae*, were reported as probiotic yeasts [24].

Culture-dependent and culture-independent methods have been applied to evaluate yeast diversity in various habitats. While culture-independent methods can be used to make a list of both culturable and unculturable yeasts, the remarkable advantage of the culture-dependent method is that pure culture is obtained, which can be used in further studies to assess medical, pharmaceutical, agricultural and industrial applications [25]. The lack of information about the yeast community in the small intestines of animals is perhaps due to the uncommon habitat that involves extreme conditions and difficulty of access. Consequently, the assessment of yeast diversity in pia and the epithelium of beef cattle small intestine was of interest. Using a culture-dependent method allows us to obtain a yeast collection that can be further characterized in terms of some useful probiotic properties, which can then be applied in industrial livestock feed in the future. Accordingly, yeast from pia and the epithelium of three parts of the beef cattle small intestine were isolated using a culture-dependent method, allowing the yeast community to then be evaluated.

## 2. Materials and Methods

### 2.1. Sample Collection

Pia and small-intestinal epithelial samples were obtained from cattle butchered at the slaughterhouse of Pon Yang Kham Livestock Breeding Cooperative N.S.C., Ltd., in Sakon Nakhon Province, Thailand (17°04′26.0″ N/104°11′53.4″ E) between August and September 2019 (Table 1). All the experiments were performed in accordance with the relevant guidelines and regulations. In addition, the animal use experiment and procedures were reviewed and approved by the Kasetsart University Institutional Animal Care and Use Committee. After the beef cattle were slaughtered, the small intestine was dissected into three parts: the duodenum, jejunum, and ileum. Then, the pia inside the small intestine was transferred to sterile centrifuge tubes, and the small intestines were kept in sterile plastic bags, sealed, and stored at 4 °C during transport to the laboratory. Then, the pH of the pia samples was measured, and yeast isolation was performed within 24 h.

### 2.2. Yeast Isolation

A culture-dependent method was used in this study. One hundred microliters of pia samples were directly spread on yeast-extract-peptone-dextrose (YPD) agar (1% yeast extract, 2% peptone, 2% dextrose and 1.5% agar), supplemented with 0.02% chloramphenicol and 0.025% sodium propionate, to inhibit bacteria and filamentous fungi, respectively. Pia samples were also directly spread onto modified medium C agar (15% pia, 0.25% yeast extract, 1% peptone, 15% solution I; 3 gL^−1^ K_2_HPO_4_, 15% solution II; 3 gL^−1^ KH_2_PO_4_, 6 gL^−1^ (NH_4_)_2_SO_4_, 6 gL^−1^ NaCl, 0.6 gL^−1^ MgSO_4_·7H_2_O, and 0.6 gL^−1^ CaCI_2_·H_2_O) [26], supplemented with 0.02% chloramphenicol and 0.025% sodium propionate. In addition, five milliliters of each pia sample were mixed with 45 mL of 0.85% NaCl solution, and the mixture was then serially diluted and spread onto YPD and modified medium C agar, supplemented with 0.02% chloramphenicol and 0.025% sodium propionate. Small intestine samples were cut using sterile techniques, and the small-intestinal epithelium was directly placed onto YPD and modified medium C agar supplemented with 0.02% chloramphenicol and 0.025% sodium propionate. All the experiments were performed in triplicates. All the plates were incubated at 35 °C until yeast colonies appeared. Different yeast colonies and cell morphologies were picked and purified on YPD agar by the cross-streak method. Subsequently, purified yeasts were preserved at −20 °C in 30% glycerol.

### 2.3. Yeast Identification

Genomic DNA was extracted from yeast cells, as previously described [27], with slight modifications. Briefly, yeast cells were suspended in 1 mL of sterile distilled water in a 1.5 mL centrifuge tube and vortexed for 5 s. Then, the upper aqueous layer was discarded. Fifty microliters of chloroform:isoamyl alcohol (24:1) were added to the tube, mixed for 5 min, and then centrifuged at 10,000× *g* for 5 min. The upper aqueous layer containing DNA was used as the DNA template in polymerase chain reactions (PCRs). The D1/D2 domain of the large subunit rRNA (LSU rRNA) gene and the internal transcribed spacer (ITS) region were amplified by PCR using the primers NL1/NL4 [28] and NS7A/NL5A [29], respectively. Then, the PCR product was visualized by 1% agarose gel electrophoresis and purified using a FavorPrepTM Gel/PCR Purification Mini Kit (Favorgen, Austria), after which, purified DNA was subjected to sequencing at First BASE Laboratories, Malaysia. BLASTn was used to compare D1/D2 and ITS sequences with those of the type strain in the GenBank database (https://www.ncbi.nlm.nih.gov/ accessed on 18 January 2021). The criteria used for ascomycetous yeast identification in this study were as follows: an isolate showing sequence alignment similarity greater than 1% (6 nucleotide substitutions) in 600 nucleotides of the D1/D2 domain was identified as a different species, and an isolate that showed nucleotide differentiation in zero to three positions was identified as a conspecific or sister species [28]. For basidiomycetous yeast identification, an isolate showing two or more nucleotide substitutions in the D1/D2 domain was identified as a different species [30]. Analysis of the ITS sequence similarity was performed when the identification by analysis of the D1/D2 domain was not clear.

### 2.4. Phylogenetic Analysis

Phylogenetic analysis was performed based on the nucleotide sequence of the D1/D2 domain of the LSU rRNA gene to confirm the yeast identification results using the MEGA (version 7.0.26) program [31]. The sequence of a representative yeast from individual species was subjected to alignment with the type strain sequence from GenBank. Then, the alignment was used for phylogenetic tree construction. The phylogenetic tree was built from the evolutionary distance using the general time reversible (GTR) model and the maximum-likelihood method. The bootstrap values were calculated from 1000 replications [32].

### 2.5. Biodiversity Analyses

The Shannon–Wiener index (*H’*) was used to characterize yeast diversity. Community evenness was determined using Shannon’s equitability (*E_H_*) [33,34]:

$$\mathrm{Shannon}–\mathrm{Wiener}\;\mathrm{index}\;(H^{\prime })=-\sum_{i=0}^{S}P_{i}\left(lnP_{i}\right)$$

$$\mathrm{SShannon}’s\;\mathrm{equitability}\;(E_{H})=(H^{\prime })/lnS$$

where *P_i_* is the proportion of each species in the sample, *S* is total number of species in the total sample, and equitability assumes a value between 0 and 1. A value approaching 1 indicates complete evenness among species, while a value approaching 0 indicates no evenness among species.

The relative frequency (%) was calculated as the number of yeast isolates of individual species divided by the total number of yeast isolates.

The frequency of occurrence (%) was calculated as the number of samples in which a particular species was observed, divided by the total number of samples.

The similarity of yeast communities associated with the pia and small-intestinal epithelium among the duodenum, jejunum, and ileum was measured using the classic Jaccard similarity coefficient. The computational analysis was performed using the PAST software version 3.25 [35]. Principal coordinate analysis (PCoA) based on Jaccard similarity indices to ordinate yeast communities in 54 samples of pia and 50 samples of the small-intestinal epithelium was performed using PAST software version 3.25 [35].

The species richness was estimated by EstimateS software, which calculated species richness from the sampling effort of the Chao 1, Jack 1 and bootstrap estimators with sample-based abundance data (classic EstimateS input) [36].

## 3. Results

### 3.1. Sample Collection and Yeast Isolation

Fifty-four pia samples, from 18 duodena, 18 jejuna, 18 ilea and 50 small-intestinal epithelial tissues from 17 duodena, 16 jejuna and 17 ilea were collected from the slaughterhouse of Pon Yang Kham Livestock Breeding Cooperative N.S.C., Ltd., in Sakon Nakhon Province, Thailand. The average pH values of the pia samples from the duodenum, jejunum, and ileum were 5.2, 7.2, and 7.8, respectively (Table 1).

A total of 1126 yeasts were isolated from pia and the small-intestinal epithelium. A total of 279, 200 and 222 yeasts were obtained from 18 (100%) duodena, 18 (100%) jejuna and 18 (100%) ileal pia samples, respectively (Table 1 and Tables S1–S3). In addition, 425 yeasts were isolated from 50 small-intestinal epithelial tissues. A total of 162 yeasts were isolated from 17 (100%) duodenal epithelial tissues, 139 yeasts were isolated from 14 (87.5%) out of 16 jejunal epithelial tissues, and 124 yeasts were isolated from 15 (88.2%) out of 17 ileal epithelial tissues (Table 1 and Tables S4–S6).

### 3.2. Identification of Yeasts from Pia

A total of 701 yeast isolates from pia were identified based on the D1/D2 domain of the LSU rRNA gene sequence (Tables S1–S3), and 695 isolates were found to be known yeast species. From the 18 duodenal pia samples, 276 isolates (98.9%) out of 279 isolates were identified as known yeast species. Among the isolates of known species, 265 isolates (96.0%) were identified as 17 species belonging to 11 genera, nine families, and two subphyla in the phylum Ascomycota: *Aureobasidium melanogenum*, *Candida ethanolica*, *Candida glabrata*, *Candida metapsilosis*, *Candida orthopsilosis*, *Candida parapsilosis*, *C. tropicalis*, *Candida vulturna*, *Cyberlindnera jadinii*, *Diutina rugosa*, *Exophiala dermatitidis*, *Lodderomyces elongisporus*, *Meyerozyma caribbica*, *Pichia kudriavzevii*, *Pichia manshurica*, *Schwanniomyces etchellsii* and *Wickerhamiella pararugosa*. The majority of these known species (15 species, 260 isolates) were in the subphylum Saccharomycotina, whereas only two species (five isolates) were in the subphylum Pezizomycotina (Table S1). The remaining 11 isolates (4.0%) of the known species were in three subphyla of the phylum Basidiomycota, i.e., *Trichosporon asahii* (one isolate) and *Trichosporon japonicum* (six isolates) in the subphylum Agaricomycotina and *Rhodotorula mucilaginosa* (four isolates) in the subphylum Pucciniomycotina (Table S1). The results revealed that yeasts in the phylum Ascomycota were the most common yeasts in duodenal pia.

From the 18 jejunal pia samples, 198 isolates (99.0%) out of 200 isolates were identified as known yeast species. Of the known species, 196 isolates (99.0%) were identified as 16 species belonging to 10 genera, nine families, and two subphyla in the phylum Ascomycota: *A. melanogenum*, *C. albicans*, *C. glabrata*, *C. metapsilosis*, *C. orthopsilosis*, *C. parapsilosis*, *C. tropicalis*, *C. vulturna*, *Cyberlindnera jadinii*, *D. rugosa*, *E. dermatitidis*, *P. kudriavzevii*, *P. manshurica*, *Sch. etchellsii*, *W. pararugosa* and *Yamadazyma olivae*. Among these known species, two species (four isolates) were in the subphylum Pezizomycotina, and 14 species (192 isolates) were in the subphylum Saccharomycotina (Table S2). Two isolates (1.0%) of known species were identified as species in the phylum Basidiomycota, including *T. japonicum* (one isolate) in the subphylum Agaricomycotina and *R. mucilaginosa* (one isolate) in the subphylum Pucciniomycotina (Table S2).

From the 18 ileal pia samples, 221 isolates (99.5%) out of 222 isolates were identified as known yeast species. From the isolates of the known species, 218 isolates (98.6%) were identified as 17 species belonging to 10 genera, nine families, and two subphyla in the phylum Ascomycota: *A. melanogenum*, *C. albicans*, *C. glabrata*, *C. metapsilosis*, *C. orthopsilosis*, *C. parapsilosis*, *C. tropicalis*, *C. vulturna*, *Cyberlindnera jadinii*, *D. rugosa*, *E. dermatitidis*, *Kodamaea ohmeri*, *M. caribbica*, *P. kudriavzevii*, *P. manshurica*, *Sch. etchellsii* and *W. pararugosa*. Of these known species, two species (six isolates) were in the subphylum Pezizomycotina, and 15 species (212 isolates) were in the subphylum Saccharomycotina (Table S3). The remaining three isolates (1.4%) were identified as three species in the phylum Basidiomycota, i.e., *T. asahii* (one isolate) and *T. japonicum* (one isolate) in the subphylum Agaricomycotina and *R. mucilaginosa* (one isolate) in the subphylum Pucciniomycotina.

The remaining six isolates among the 701 pia isolates exhibited greater than 1% nucleotide substitution in the D1/D2 region of the LSU rRNA gene, as compared to the most closely related species. Therefore, the taxonomic placement of these six isolates should be clarified. According to pairwise sequence analysis using BLASTn, two isolates (PYD 90-26 and PYD 90-28) were closely related to *Wickerhamiella infanticola*, and four isolates (PCJ 90-11, PCJ 90-16, PYI 91-12 and PYD 99-2) were closely related to *Cyberlindnera jadinii*. These isolates will therefore be characterized and proposed as novel yeast species.

### 3.3. Comparison of Yeasts from Pia of Three Parts of the Small Intestine

Yeasts could be isolated from all the pia samples from all three small-intestinal parts, and the yeast community was described by the Shannon–Weiner index (*H’*) and equitability (*E_H_*). The *H’* of the yeast community in the pia of the jejuna and ilea were 2.3 and 2.4, respectively, while the *E_H_* of both was 0.8, showing greater diversity than the duodenal pia, in which the *H’* and *E_H_* were 2.2 and 0.8, respectively (Table 2).

**Figure 1:**
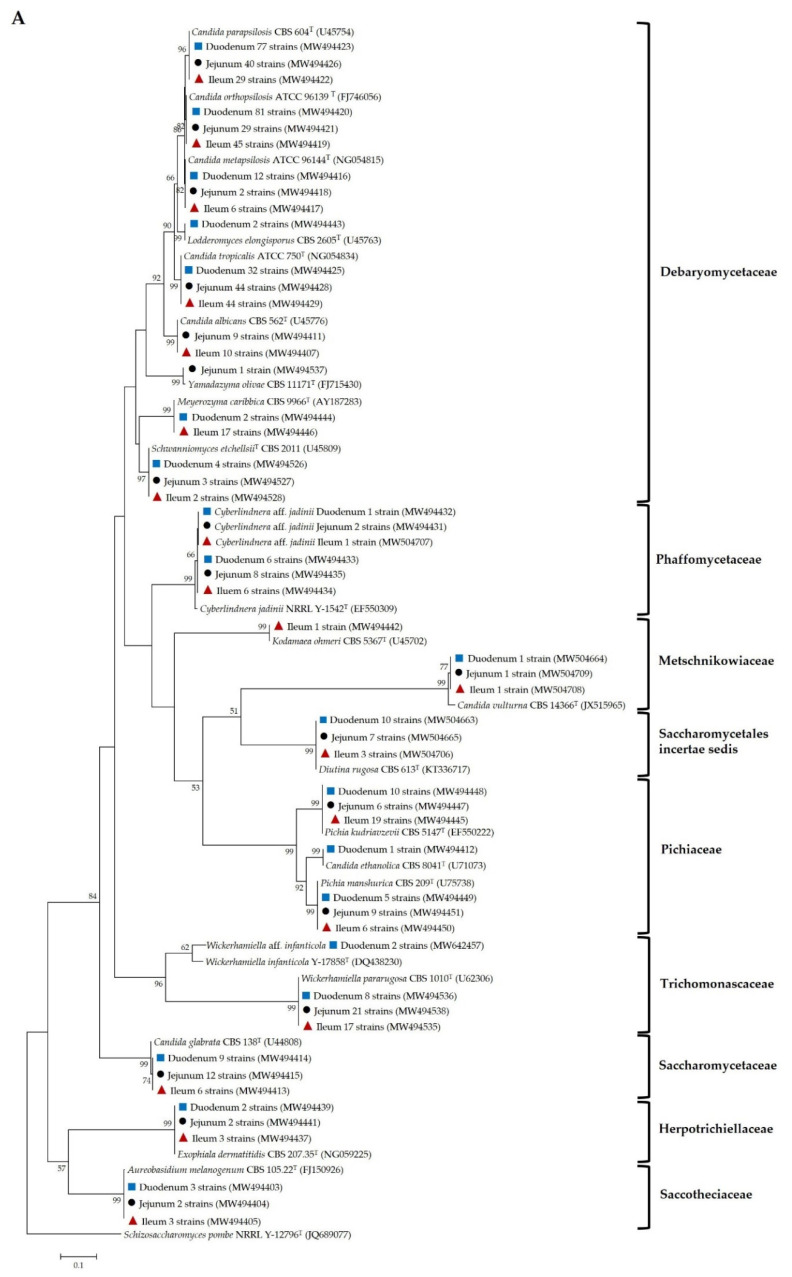

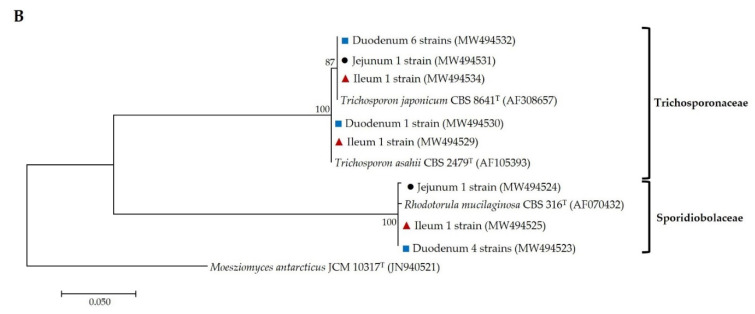
Phylogenetic placement based on the sequence analysis of the D1/D2 domain of the LSU rRNA gene using the maximum-likelihood method (GTR model) of representative yeast species isolated from duodenal pia (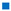), jejunal pia (●) and ileal pia (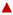) and their closely related type strain sequences received from the GenBank database. Numbers on branches demonstrate the bootstrap percentages (>50%), derived from 1000 replications. (**A**) Phylum Ascomycota; scale bar, 0.1 substitutions per nucleotide position; *Schizosaccharomyces pombe* NRRL Y-12796^T^ (JQ689077) was used as the outgroup; (**B**) Phylum Basidiomycota; scale bar, 0.05 substitutions per nucleotide position; *Moesziomyces antarcticus* JCM 10317^T^ (JN940521) was used as the outgroup.

The relative frequency (RF) and frequency of occurrence (FO) of yeast species obtained from the duodenal, jejunal and ileal pia are compared in Table 3. Moreover, phylogenetic placement of the representative yeast isolates from pia was also performed to reveal the relationships of yeast taxa (Figure 1). The results revealed that the common yeast species found in duodenal, jejunal and ileal pia belonged to Ascomycota. In addition, the majority of ascomycetous yeasts in pia did not differ among the three parts and were identified as belonging to Saccharomycotina (Debaryomycetaceae). *C. parapsilosis* was the most prevalent species in the duodenal and jejunal pia, with frequencies of occurrence (FOs) of 83.3% and 77.8%, respectively, while the relative frequencies were 27.6% and 20.0%, respectively. Interestingly, both *C. parapsilosis* and *C. tropicalis* were the prevalent species in the ileal pia, with a 61.1% FO and a 13.1% and 19.8% RF, respectively.

*A. melanogenum*, *C. glabrata*, *C. metapsilosis*, *C. orthopsilosis*, *C. parapsilosis*, *C. tropicalis*, *C. vulturna*, *Cyberlindnera jadinii*, *D. rugosa*, *E. dermatitidis*, *P. kudriavzevii*, *P. manshurica*, *R. mucilaginosa*, *Sch. etchellsii*, *T. japonicum*, *W. pararugosa* and *Y. olivae* were discovered in the yeast community of pia collected from all three parts of the small intestine. *C. albicans* was obtained from the jejunum and ileum, whereas *M. caribbica* and *T. asahii* were present in the duodenum and ileum. However, *C. ethanolica* and *L. elongisporus* were found only in the duodenum. *Y. olivae* and *K. ohmeri* were only detected in the jejunum and ileum, respectively.

**Figure 2:**
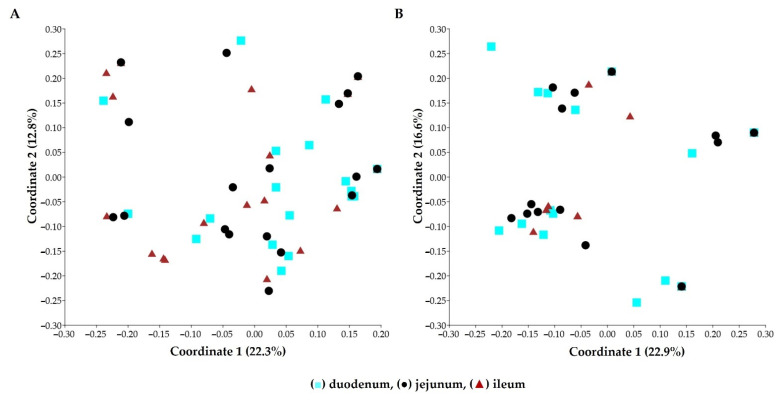
Principal coordinate analysis (PCoA) plots of yeast communities in (**A**) 54 pia samples and (**B**) 50 small-intestinal epithelial samples of the duodenum, jejunum, and ileum using the Jaccard similarity coefficient.

**Figure 3:**
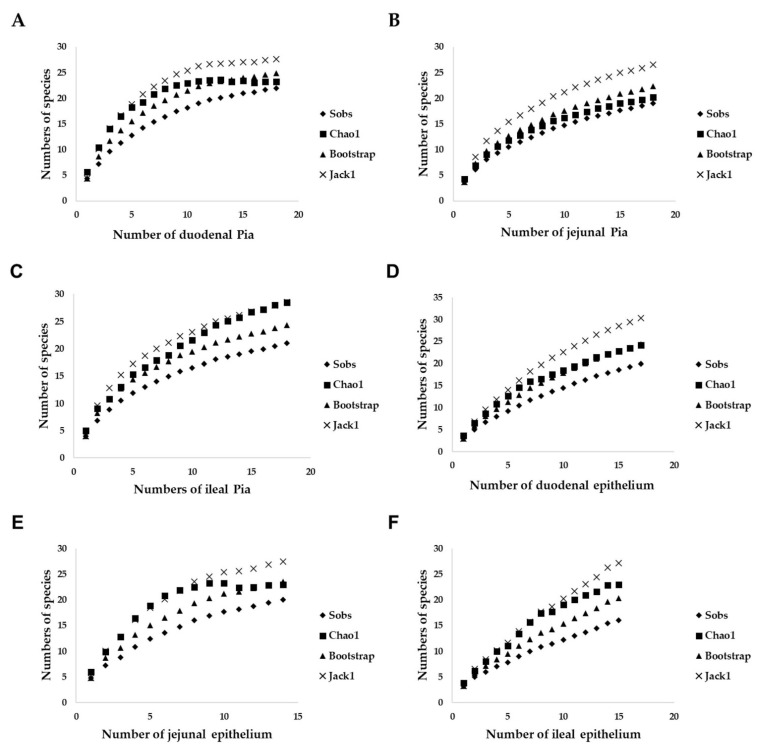
Species accumulation curves showing the relationship between the number of pia or epithelial samples and the number of species observed (sobs). Chao 1, Jack 1 and bootstrap species richness estimators were plotted: (**A**) yeasts from duodenal pia, (**B**) yeasts from jejunal pia, (**C**) yeasts from ileal pia, (**D**) yeasts from duodenal epithelium, (**E**) yeasts from jejunal epithelium, and (**F**) yeasts from ileal epithelium.

Principal coordinate analysis (PCoA) plots indicated that there were no marked differences in the yeast communities in pia from three parts of the intestine (Figure 2A). Nevertheless, estimation of the expected richness of species showed that the observed species richness was lower than predicted in all three parts, which indicated that unobserved yeast species remained in the pia in this study (Figure 3A–C).

### 3.4. Identification of Yeasts from the Small-Intestinal Epithelium

A total of 425 yeast isolates, which were obtained from the epithelium of three small-intestinal parts, were identified using the D1/D2 region of the LSU rRNA gene sequence (Tables S4–S6).

From the 17 duodenal epithelial samples, 160 isolates (98.8%) out of 162 isolates were identified as known yeast species. Among the isolates of known species, 142 isolates (87.7%) were identified as 16 known species belonging to 10 genera, seven families, and two subphyla in the phylum Ascomycota, namely, *Candida duobushaemulonii*, *C. metapsilosis*, *C. orthopsilosis*, *C. parapsilosis*, *C. tropicalis*, *C. vulturna*, *C. zeylanoides*, *Debaryomyces hansenii*, *D. rugosa*, *E. dermatitidis*, *Kluyveromyces marxianus*, *M. caribbica*, *P. manshurica*, *Sch. etchellsii*, *W. pararugosa* and *Yarrowia lipolytica*. Among these known yeast species, one species (one isolate) was in the subphylum Pezizomycotina, and 15 species (141 isolates) were in the subphylum Saccharomycotina (Table S4). In addition, 18 isolates (11.1%) out of 160 known species were identified as species in the phylum Basidiomycota, including *T. asahii* (one isolate) and *T. japonicum* (three isolates) in the subphylum Agaricomycotina and *R. mucilaginosa* (14 isolates) in the subphylum Pucciniomycotina (Table S4).

From the 16 jejunal epithelial samples, all 139 isolates were identified as known yeast species. Of the known yeast species, 120 isolates (86.3%) were identified as 18 species belonging to 10 genera, seven families, and two subphyla in the phylum Ascomycota, i.e., *C. duobushaemulonii*, *C. metapsilosis*, *C. orthopsilosis*, *C. parapsilosis*, *C. tropicalis*, *C. vulturna*, *C. zeylanoides*, *D. hansenii*, *D. rugosa*, *E. dermatitidis*, *Kluyveromyces marxianus*, *K. ohmeri*, *P. kudriavzevii*, *P. manshurica*, *Sch. etchellsii*, *W. infanticola*, *W. pararugosa* and *Y. olivae*. Among these known species, only one species (one isolate) was in the subphylum Pezizomycotina, and 17 species (119 isolates) were in the subphylum Saccharomycotina (Table S5). In addition, 19 isolates (13.7%) of known species were identified as species in the phylum Basidiomycota, i.e., *T. japonicum* (two isolates) in the subphylum Agaricomycotina and *R. mucilaginosa* (17 isolates) in the subphylum Pucciniomycotina.

For the 17 ileal epithelial samples, 124 isolates were obtained, and all of them were identified as known yeast species. One hundred and seven isolates (86.3%) out of 124 isolates were identified as 13 species belonging to eight genera and six families in the subphylum Saccharomycotina of the phylum Ascomycota, i.e., *C. albicans*, *C. duobushaemulonii*, *C. orthopsilosis*, *C. parapsilosis*, *C. tropicalis*, *C. zeylanoides*, *Clavispora lusitaniae*, *D. rugosa*, *Kluyveromyces marxianus*, *P. kudriavzevii*, *P. manshurica*, *Sch. etchellsii* and *W. pararugosa.* (Table S6). In addition, 17 isolates (13.7%) of known species belonged to the phylum Basidiomycota, i.e., *T. japonicum* (one isolate) in the subphylum Agaricomycotina and *Sterigmatomyces elviae* (one isolate) and *R. mucilaginosa* (15 isolates) in the subphylum Pucciniomycotina.

Nevertheless, two isolates out of 425 isolates from the intestinal epithelium showed nucleotide substitutions greater than 1% in the D1/D2 domain of the LSU rRNA gene with their closest species. Therefore, these two isolates should be examined further to determine whether they are novel or known yeasts. According to pairwise sequence analysis using BLASTn, the two isolates (CTD 88-2 and CTD 100-5) are closely related to *Wickerhamiella infanticola*, and they are characterized and proposed as novel species.

### 3.5. Comparison of Yeasts Obtained from the Small-Intestinal Epithelium in All Three Parts

Yeasts could be isolated from 46 small-intestinal epithelial samples (92%). The yeast community was assessed by the Shannon–Weiner index (*H’*) and equitability (*E_H_*). The yeast community in the jejunal epithelium was the most diverse, with an *H’* of 2.0 and an *E_H_* of 0.7, while the *H’* values of the yeast communities of the duodena and ilea were 1.8 and 1.7, respectively, and the *E_H_* values of the yeast communities of the duodena and ilea were similar at 0.6 (Table 2).

**Figure 4:**
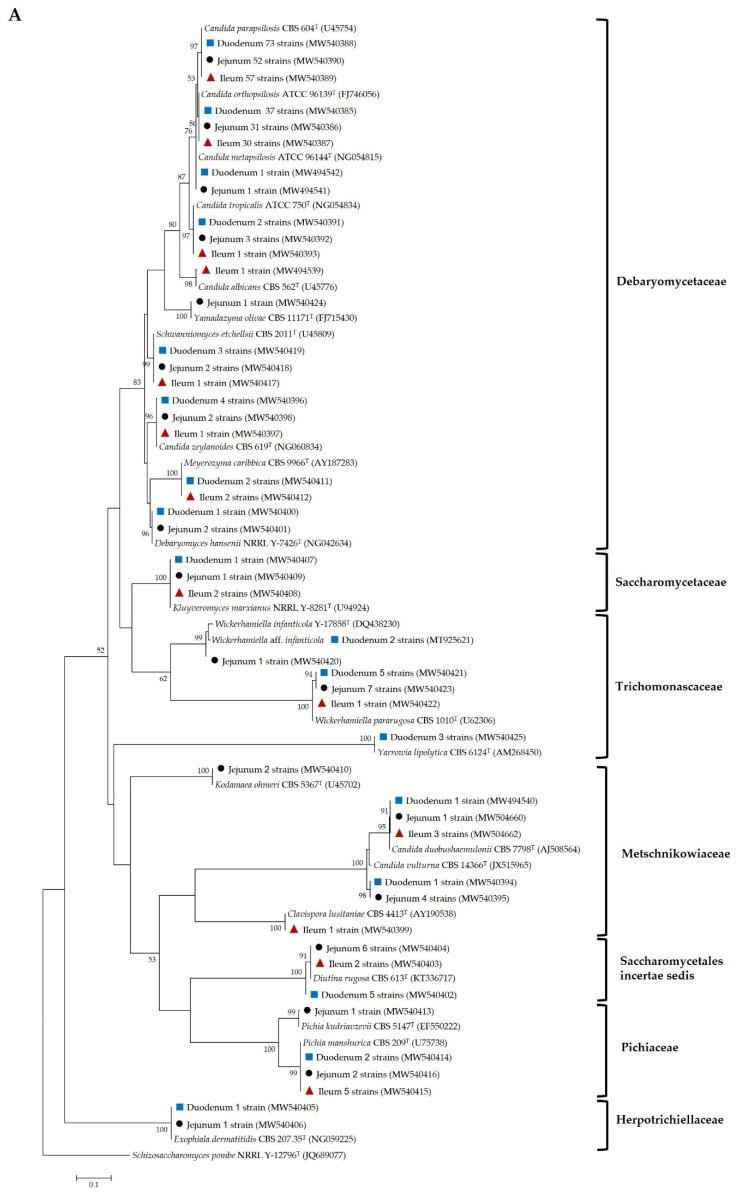

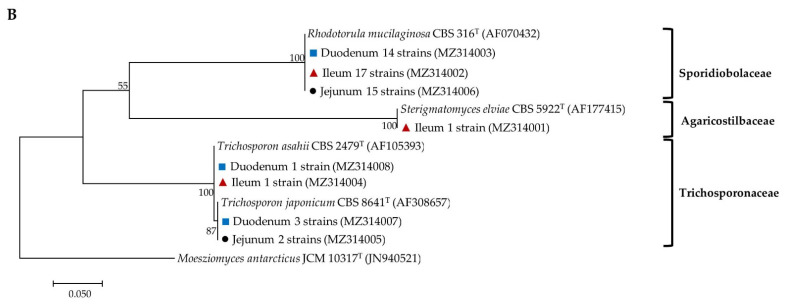
Phylogenetic placement based on the sequence analysis of the D1/D2 domain of the LSU rRNA gene using the maximum-likelihood method (GTR model) of representative yeast species isolated from the small-intestinal epithelium of duodena (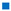), jejuna (●) and ilea (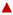) and their closely related type strain sequences received from the GenBank database. Numbers on branches demonstrate the bootstrap percentages (>50%), derived from 1000 replications. (**A**) Phylum Ascomycota; scale bar, 0.1 substitutions per nucleotide position; *Schizosaccharomyces pombe* NRRL Y-12796^T^ (JQ689077) was used as the outgroup; (**B**) phylum basidiomycota; scale bar, 0.05 substitutions per nucleotide position; *Moesziomyces antarcticus* JCM 10317^T^ (JN940521) was used as the outgroup.

The comparison of the relative frequency (RF) and frequency of occurrence (FO) of yeast species obtained from epithelia of the three parts of the small intestine is shown in Table 4. Furthermore, phylogenetic placement of representative isolated yeasts from the small-intestinal epithelium was also constructed to compare yeast taxa (Figure 4). The results showed that the common yeast species discovered in the epithelium of all three parts of the small intestine belonged to Ascomycota. In addition, the majority of ascomycetous yeasts in the epithelium of all three parts of the small intestine were not different and belonged to the phylum Saccharomycotina (Debaryomycetaceae). Interestingly, the most prevalent yeast species in the small-intestinal epithelium of the duodenum, jejunum, and ileum was *C. parapsilosis*, with FO values of 88.2%, 87.5%, and 87.2%, respectively, and RF values of 45.1%, 37.4% and 46.0%, respectively.

Among the yeasts obtained from the small-intestinal epithelium, *C. duobushaemulonii*, *C. orthopsilosis*, *C. parapsilosis*, *C. tropicalis*, *C. zeylanoides*, *D. rugosa*, *Kluyveromyces marxianus*, *P. manshurica*, *R. mucilaginosa*, *Sch. etchellsii* and *W. pararugosa* were found in all three parts of the small intestine. *C. metapsilosis*, *C. vulturna*, *D. hansenii*, *E. dermatitidis* and *T. japonicum* were obtained from the duodenal and jejunal epithelia, whereas *M. caribbica* and *T. asahii* were isolated from the duodenal and ileal epithelia. However, Yarrowia lipolytica and *K. ohmeri* were only found in the duodenal epithelium and jejunal epithelium, respectively. *C. albicans*, *C. lusitaniae* and *S. elviae* were only found in the ileal epithelium.

No marked differences were found in yeast communities from the epithelium of any of the three parts of the small intestine, as indicated by principal coordinate analysis (PCoA) (Figure 2B). Nonetheless, estimation of the expected richness of species showed that the observed species richness was lower than predicted in all three parts, which indicated that unobserved yeast species remained in the small-intestinal epithelium in this study (Figure 3D–F).

### 3.6. Comparison of the Yeast Communities of the Small-Intestinal Pia and Epithelium

In terms of species richness, the yeast community was more diverse in the pia of the duodenum, jejunum, and ileum (indicated by *H’* indices of 2.2, 2.3, and 2.4, respectively) than in the epithelium of the duodenum, jejunum, and ileum (indicated by *H’* indexes of 1.8, 2.0, and 1.7, respectively), whereas the yeast species evenness, *E_H_*, in the duodenal, jejunal, and ileal pia (0.7, 0.8, and 0.8, respectively) was similar to that in the epithelium of the duodenum, jejunum, and ileum (0.6, 0.7, and 0.6, respectively) (Table 2). The relative frequency (RF) and frequency of occurrence (FO) of yeast species obtained from the pia and epithelium are shown in Table 3 and Table 4, respectively. The results indicated that the common yeast species obtained from both pia and the epithelium of all three parts of the small intestine belonged to Ascomycota. Moreover, the majority of ascomycetous yeast found in both the pia and epithelium of all three parts were not different and belonged to the phylum Saccharomycotina (Debaryomycetaceae). Notably, the yeast species with the highest occurrence obtained from both the pia and epithelium was *Candida parapsilosis*. The FOs of *C. parapsilosis* found in the duodenal, jejunal and ileal pia were 83.3%, 77.8% and 61.1%, respectively, while the RF values were 27.6%, 20.0% and 13.1%, respectively. In addition, the FOs of *C. parapsilosis* in the duodenal, jejunal and ileal epithelia was 88.2%, 87.5% and 88.2%, respectively, whereas the RF values were 45.1%, 37.4% and 46.0%, respectively. However, *C. tropicalis* was also the yeast species with the highest occurrence in the pia of the ileum, for which the OF and RF were 61.1% and 19.8%, respectively.

The yeasts *C. albicans*, *C. metapsilosis*, *C. orthopsilosis*, *C. parapsilosis*, *C. tropicalis*, *C. vulturna*, *D. rugosa*, *E. dermatitidis*, *K. ohmeri*, *M. caribbica*, *P. kudriavzevii*, *P. manshurica*, *R. mucilaginosa*, *Sch. etchellsii*, *T. asahii*, *T. japonicum*, *W. pararugosa* and *Y. olivae* were found in both the pia and epithelium. On the other hand, *A. melanogenum*, *C. ethanolica*, *C. glabrata*, *Cyberlindnera jadinii* and *L. elongisporus* were found in only the pia, while *C. duobushaemulonii*, *C. zeylanoides*, *Clavispora lusitaniae*, *Debaryomyces hansenii*, *Kluyveromyces marxianus*, *Sterigmatomyces elviae*, *W. infanticola* and *Yarrowia lipolytica* were only found in the epithelium.

## 4. Discussion

Evaluation of yeast diversity has been reported in the digestive tract of animal species. However, the present study is the first to report yeast community and diversity in the internal contents and epithelium of the small intestine of beef cattle. The small intestine is located in the digestive tract next to the stomach, the internal contents of which are highly acidic [37]. The pH of the pia samples was therefore measured, and the results showed that the average pH values were 5.9, 7.2 and 7.8 in the pia of the duodenum, jejunum, and ileum, respectively. Other studies also reported that the pH values of each part of the ruminant small intestine differs considerably [38,39,40], especially between the beginning of the duodenum and the terminus of the ileum, because the chyme that enters the duodenum (pH 2.5) is mixed with pancreatic juice (pH 8) [37,41] and the pH increases along the intestine. It is of interest to assess the yeast community in the small intestine of beef cattle since general yeast growth was found to be optimal at pH 4.5 to 5.5; however, some yeast species, such as *P. kudriavzevii* and *Pichia membranifaciens*, that are isolated from spoiled food could tolerate pH values of 1.3 to 1.7 [42]. In contrast, the work in [43] reported that many genera of yeasts, such as *Lodderomyces*, *Trichosporon* and *Sterigmatomyces*, showed alkaline-tolerance properties. Some yeasts, such as *Dabaryomyces hansenii*, also grew under alkaline conditions because protons were pumped out of the cell by a plasma membrane ATPase and weak acids were produced to increase the level of protons within the cell [44]. However, another limiting factor for the growth of some yeasts is secondary bile acid. The growth of *C. albicans* cells, as well as germ tube, hypha and biofilm formation, were inhibited by lithocholic acid (LCA) and deoxycholic acid (DCA) [45]. While pH and bile acid affect the subsistence of yeasts in the small intestine, some yeasts still survive and grow under these conditions. Therefore, it would be interesting to assess the probiotic properties of the yeast species discovered in pia in further studies.

In this work, the occurrence of yeasts associated with the duodenal, jejunal and ileal pia appeared to be similar in terms of yeast taxa. *C. parapsilosis*, *C. tropicalis*, *C. glabrata* and *P. kudriavzevii* (formerly *C. krusei*), which were found in the pia of all three parts of the small intestine in this study, were also found in the bovine cecum [20]. Interestingly, *C. tropicalis* was the most prevalent species in the ileal pia and was also the most dominant species in the bovine cecum. Additionally, Van Uden et al. [21] studied yeasts in the cecum of horses, sheep, goats and swine, and the common yeasts recovered from each animal were *Trichosporon cutaneum*, *C. albicans*, *P. kudriavzevii* and *C. slooffii*. In contrast, *C. parapsilosis* was the most common species in both the pia and small-intestinal epithelium of the duodenum, jejunum, and ileum of cattle in this work. While *C. parapsilosis* was not dominant, this species was also found in the cecum of all animals, except for goats. The occurrence of yeasts was observed in some poultry. The predominant yeast obtained from the cloacae of migratory birds was *Rhodotorula rubra* (28.2%), followed by *Cryptococcus albidus* (18.4%), *C. albicans* (9.2%), *T. cutaneum* (8.4%), *C. guilliermondii* (6.1%) and *C. tropicalis* (6.1%) [46]. In addition, the prevalent yeast in the gastrointestinal tract of healthy turkeys was *C. catenulata* (30.7%), followed by *C. albicans* (21.7%), *C. palmioleophila* (17.4%), *D. rugosa* (17.4%), and *C. glabrata* (8.7%). This result indicated that some yeast species reported in poultries—i.e., *C. albicans*, *C. tropicalis*, *D. rugosa* and *C. glabrata*—could also be found in the pia and small-intestinal epithelium.

Yeast communities have been evaluated in several different ecosystems. In 1974, Lund [47] isolated yeast from bovine rumen fluid and showed that *P. kudriavzevii, T. cutaneum* and *T. capitatum* were the predominant species. Recently, Fernandes et al. [18] isolated yeasts from bovine rumen fluid, and the major yeast species were found to be *P. kudriavzevii, D. rugosa, W. pararugosa* and *C. ethanolica*. Interestingly, all the predominant yeast species found in bovine rumen fluid were also observed in the present work, with the exception of *T. cutaneum* and *T. capitatum*. However, different members of *Trichosporon*, i.e., *T. asahii* and *T. japonicum* were found. *T. asahii* was only obtained from pia and the epithelium of the duodenum and the ileum, whereas *T. japonicum* was discovered from pia of all parts and from the duodenal and jejunal epithelia. The results indicate that these yeast species may be common in the gastrointestinal tract of cattle.

Currently, both culture-dependent and culture-independent approaches have been employed to assess yeast communities. The study of the yeast community in traditional cheese using both approaches revealed that *Debaryomyces hansenii* and *Kluyveromyces marxianus* were the main yeast species when evaluated by the culture-dependent method, while the culture-independent method showed that *Debaryomyces*, *Kluyveromyces* and *Galactomyces* were the main yeast genera [48]. A study of the fungal community in the stem of grapevine plants (*Vitis vinifera*) showed that *Alternaria alternata* and members of the genus *Cladosporium,* including *Cladosporium cladosporioides*, *C. ramotenellum*, *C. silenes*, *C. sphaerospermum*, *C. tenellum* and *C. tenuissimum*, were the most frequently detected fungi using the culture-dependent method, whereas fungi in the family Pleosporaceae and the genera *Cladosporium*, *Botryosphaeria* and *Codophora* were reported as the most commonly detected taxa using the culture-independent method [49]. In addition, Hamad et al. [50] studied fungal population in the human gut, which indicated that *Aspergillus niger*, *Candida glabrata*, *C. parapsilosis*, *Clavispora lusitaniae*, *Debaryomyces hansenii* and *C. albicans* were the most commonly isolated fungi, while the genera *Torulaspora*, *Debaryomyces*, *Aureobasidium*, *Aspergillus*, *Penicillium* and the species *C. albicans*, *Kluyveromyces marxianus*, *Saccharomyces cerevisiae* and *Malassezia globose* were found to be the most frequently detected taxa using the culture-independent method. All these previous studies indicate that the culture-dependent method revealed narrow fungal communities, as compared to those revealed using the cultured-independent method. However, it should be noted that, when the culture-dependent method was used, the isolated strains could be identified accurately to the species level. However, when the culture-independent method was used, the higher taxa were mostly identified. In addition, microbial stains collection, which can be extensively studied, were obtained using the culture-dependent method.

Several yeast genera discovered in this work have been reported as probiotics that improve the nutrition and health of animals, including *Pichia*, *Yarrowia*, *Candida*, *Debaryomyces* and *Kluyveromyces* [24]. In contrast, although some yeast genera are also frequently found in healthy humans and animals and are considered commensal [51,52,53,54,55], they also cause diseases in humans and animals, such as *C. albicans*, *C. glabrata*, *C. parapsilosis* and *C. tropicalis*, which are prominent opportunistic pathogens that cause candidiasis in humans and animals [56,57,58,59,60]. Likewise, *Trichosporon* species are usually found in soil and are a part of the normal flora of human skin and the gastrointestinal tract of humans [61], but members of this genus have also been described as opportunistic pathogens in immunocompromised patients [62,63]. However, the yeasts obtained in this work were isolated from healthy cattle; therefore, some beneficial characteristics of the isolated yeasts will be explored to support animal health in the future.

## 5. Conclusions

In this study, we presented, for the first time, the occurrence and community of yeast in pia, which is used to treat sick cattle and for cooking in several traditional dishes in the northern and northeastern regions of Thailand. In addition, yeasts associated with the small-intestinal epithelium were also assessed based on a culture-dependent approach. The results revealed that ascomycetous yeasts were the dominant yeasts in both pia and the small-intestinal epithelium of all three parts. Moreover, ascomycetous yeasts, which belong to the family Debaryomycetaceae, constituted the majority of the yeasts in all the samples. *C. parapsilosis* was the most common yeast species in both the pia and small-intestinal epithelium. However, to comprehensively elucidate the yeast community in this area, we suggest the use of a culture-independent method, together with a culture-dependent method. In addition, some yeast species isolated in this work will be interesting to evaluate for application as probiotic yeasts in further research.

## Figures and Tables

**Table 1:** Number of yeast isolates isolated from pia and small-intestinal epithelium samples collected from the slaughterhouse of Pon Yang Kham Livestock Breeding Cooperative N.S.C., Ltd., in Sakon Nakhon Province, Thailand.

Sample Type	Sample Site	Average pH Value	Number of Samples	No. of Yeast Isolates
Pia	Duodenum	5.9	18	279
	Jejunum	7.2	18	200
	Ileum	7.8	18	222
		Total	54	701
Epithelium	Duodenum	-	17	162
	Jejunum	-	16	139
	Ileum	-	17	124
		Total	50	425

**Table 2:** Diversity indices of yeasts from the pia and epithelium from all three parts of the small intestine.

Diversity Indices	Duodenum		Jejunum		Ileum	
	Pia	Epithelium	Pia	Epithelium	Pia	Epithelium
Total number of collected samples	18	17	18	16	18	17
Total number of samples obtained yeasts	18	17	18	14	18	15
Total number of yeast isolates	279	162	200	139	222	124
Total number of yeast species (S)	22	20	19	20	21	16
Shannon-Weiner index (*H’*)	2.2	1.8	2.3	2.0	2.4	1.7
Equitability index (*E_H_*)	0.7	0.6	0.8	0.7	0.8	0.6
The most prevalent known yeast species	*C. parapsilosis*	*C. parapsilosis*	*C. parapsilosis*	*C. parapsilosis*	*C. parapsilosis*, *C. tropicalis*	*C. parapsilosis*

**Table 3:** Relative frequency and frequency of occurrence of yeasts isolated from pia samples.

Yeast Taxa	Relative Frequency (%) ^1^/Frequency of Occurrence (%) ^2^		
	Duodenum	Jejunum	Ileum
**Ascomycota**			
**Pezizomycotina**			
**Herpotrichiellaceae**			
* Exophiala dermatitidis*	0.7/11.1	1.0/5.6	1.4/11.1
**Saccotheciaceae**			
* Aureobasidium melanogenum*	1.1/5.6	1.0/5.6	1.4/5.6
**Saccharomycotina**			
**Debaryomycetaceae**			
* Candida albicans*	nd	4.5/11.1	4.5/11.1
* Candida metapsilosis*	4.3/38.9	1.0/11.1	2.7/22.2
* Candida orthopsilosis*	29.0/66.7	14.5/50.0	20.3/55.6
* Candida parapsilosis*	27.6/83.3	20.0/77.8	13.1/61.1
* Candida tropicalis*	11.5/38.9	22.0/50.0	19.8/61.1
* Lodderomyces elongisporus*	0.7/11.1	nd	nd
* Meyerozyma caribbica*	0.7/11.1	nd	7.7/5.6
* Schwanniomyces etchellsii*	1.4/16.7	1.5/5.6	0.9/11.1
* Yamadazyma olivae*	nd	0.5/5.6	nd
**Metschnikowiaceae**			
* Candida vulturna*	0.4/5.6	0.5/5.6	0.5/5.6
* Kodamaea ohmeri*	nd	nd	0.5/5.6
**Phaffomycetaceae**			
* Cyberlindnera* aff. *jadinii*	0.4/5.6	1.0/5.6	0.5/5.6
* Cyberlindnera jadinii*	2.2/11.1	4.0/22.2	2.7/22.2
**Pichiaceae**			
* Candida ethanolica*	0.4/5.6	nd	nd
* Pichia kudriavzevii*	3.6/16.7	3.0/11.1	8.6/22.2
* Pichia manshurica*	1.8/16.7	4.5/27.8	2.7/27.8
**Saccharomycetaceae**			
* Candida glabrata*	3.2/16.7	6.0/16.7	2.7/16.7
**Saccharomycetales** incertae sedis			
* Diutina rugosa*	3.6/33.3	3.5/16.7	1.4/16.7
**Trichomonascaceae**			
* Wickerhamiella aff. infanticola*	0.7/5.6	nd	nd
* Wickerhamiella pararugosa*	2.9/27.8	10.5/38.9	7.7/44.4
**Basidiomycota**			
**Agaricomycotina**			
**Trichosporonaceae**			
* Trichosporon asahii*	0.4/5.6	nd	0.5/5.6
* Trichosporon japonicum*	2.2/16.7	0.5/5.6	0.5/5.6
**Pucciniomycotina**			
**Sporidiobolaceae**			
* Rhodotorula mucilaginosa*	1.4/16.7	0.5/5.6	0.5/5.6

^1^ Relative frequency (%) was calculated as the number of isolates of a particular species as a proportion of the total number of isolates. ^2^ Frequency of occurrence (%) was calculated as number of samples, where a particular species was observed as a proportion of the total number of samples.

**Table 4:** Relative frequency and frequency of occurrence of yeasts isolated from small-intestinal epithelium.

Yeast Taxa	Relative Frequency (%) ^1^/Frequency of Occurrence (%) ^2^		
	Duodenum	Jejunum	Ileum
**Ascomycota**			
**Pezizomycotina**			
**Herpotrichiellaceae**			
* Exophiala dermatitidis*	0.6/5.9	0.7/6.3	nd
**Saccharomycotina**			
**Debaryomycetaceae**			
* Candida albicans*	Nd	nd	0.8/5.9
* Candida metapsilosis*	0.6/5.9	0.7/6.3	nd
* Candida orthopsilosis*	22.8/58.8	22.3/56.3	24.2/58.8
* Candida parapsilosis*	45.1/88.2	37.4/87.5	46.0/88.2
* Candida tropicalis*	1.2/11.8	2.2/18.8	0.8/5.9
* Candida zeylanoides*	2.5/5.9	1.4/6.3	0.8/5.9
* Debaryomyces hansenii*	0.6/5.9	1.4/12.5	nd
* Meyerozyma caribbica*	1.2/5.9	nd	1.6/5.9
* Schwanniomyces etchellsii*	1.9/17.6	1.4/12.5	0.8/5.9
* Yamadazyma olivae*	nd	0.7/6.3	nd
**Metschnikowiaceae**			
* Candida duobushaemulonii*	0.6/5.9	0.7/6.3	2.4/5.9
* Candida vulturna*	0.6/5.9	2.9/18.8	nd
* Clavispora lusitaniae*	nd	nd	0.8/5.9
* Kodamaea ohmeri*	nd	1.4/12.5	nd
**Pichiaceae**			
* Pichia kudriavzevii*	nd	0.7/6.3	nd
* Pichia manshurica*	1.2/5.9	1.4/12.5	4.0/5.9
**Saccharomycetaceae**			
* Kluyveromyces marxianus*	0.6/5.9	0.7/6.3	1.6/5.9
**Saccharomycetales incertae sedis**			
* Diutina rugosa*	3.1/11.8	4.3/25	1.6/11.8
**Trichomonascaceae**			
* Wickerhamiella* aff. *infanticola*	1.2/11.8	nd	nd
* Wickerhamiella infanticola*	nd	0.7/6.3	nd
* Wickerhamiella pararugosa*	3.1/23.5	5.0/18.8	0.8/5.9
* Yarrowia lipolytica*	1.9/11.8	nd	nd
**Basidiomycota**			
**Agaricomycotina**			
**Trichosporonaceae**			
* Trichosporon asahii*	0.6/5.9	nd	0.8/5.9
* Trichosporon japonicum*	1.9/5.9	1.4/12.5	nd
**Pucciniomycotina**			
**Agaricostilbaceae**			
* Sterigmatomyces elviae*	nd	nd	0.8/5.9
**Sporidiobolaceae**			
* Rhodotorula mucilaginosa*	8.6/52.9	12.2/43.8	12.1/52.9

^1^ Relative frequency (%) was calculated as the number of isolates of a particular species as a proportion of the total number of isolates. ^2^ Frequency of occurrence (%) was calculated as number of samples, where a particular species was observed as a proportion of the total number of samples.

## Supplementary Materials

The following are available online at https://www.mdpi.com/article/10.3390/microorganisms9071444/s1. Table S1: Identification of yeast from duodenal pia samples based on the D1/D2 domain sequence analysis, Table S2: Identification of yeast from jejunal pia samples based on the D1/D2 domain sequence analysis, Table S3: Identification of yeast from ileal pia samples based on the D1/D2 domain sequence analysis, Table S4: Identification of yeast from duodenal epithelium samples based on the D1/D2 domain sequence analysis, Table S5: Identification of yeast from jejunal epithelium samples based on the D1/D2 domain sequence analysis, Table S6: Identification of yeast from ileal epithelium samples based on the D1/D2 domain sequence analysis.
