# Supplementary material for: Yeasts Associated with the Small-Intestinal Contents and Epithelium of Pon Yang Kham (Charolais Crossbred) Fattening Beef Cattle
Source: Microorganisms. 2021 Jul 4;9(7):1444. doi: 10.3390/microorganisms9071444 (PMC8305955; doi:10.3390/microorganisms9071444)
Supplement: Supplementary file 1 [file microorganisms-09-01444-s001.zip › microorganisms-1260266-supplementary.pdf]

Supplementary Material

Table S1: Identification of yeast from duodenal Pia samples based on the D1/D2 domain sequence analysis.

Taxa	No. of strain	strain	D1/D2 GenBank accession no. of closest species	Nucleotide substitutions to closest species	NO.of samples
**Ascomycota**					
**Pezizomycotina**					
Herpotrichiellaceae					
Exophiala dermatitidis	2	PYD3-1, PYD93-25	NG059225	0	2
Saccotheciaceae					
Aureobasidium elanogenum	3	PYD95-7, PYD95-13, PYD95-14	FJ150926	0,3	1
**Saccharomycotina**					
Debaryomycetaceae					
Candida metapsilosis	12	PCD1-1, PCD3-8, PYD88-14, PYD88-15, PCD88-2, PCD92-3, PYD93-8, PCD93-17, PCD93-20, PYD95-3, PYD96-15, PYD96-21	NG054815	0-1	7
Candida orthopsilosis	81	PYD1-6, PYD3-2, PYD3-8, PCD3-1, PCD3-2, PCD3-7, PYD6-3, PYD88-3, PYD88-13, PCD88-3, PCD88-6, PCD89-2, PCD90-3, PCD90-21, PCD90-26, PYD91-3, PYD91-6, PYD91-7, PYD93-1, PYD93-2, PYD93-4, PYD93-9, PYD93-12, PYD93-15, PYD93-18, PYD93-20, PYD93-21, PYD93-23, PYD93-24, PYD93-27, PCD93-1, PCD93-2, PCD93-3, PCD93-4, PCD93-6, PCD93-7, PCD93-8, PCD93-9, PCD93-10, PCD93-12, PCD93-13, PCD93-14, PYD95-2, PYD95-5, PYD95-9, PYD95-10, PYD95-12, PYD95-15, PYD95-16, PYD95-17, PYD95-19, PCD95-1, PCD95-3, PCD95-5, PCD95-10, PCD95-12, YD96-1, PYD96-2, PYD96-5, PYD96-7, PYD96-9,	FJ746056	0-1	12

Taxa	No. of strain	strain	D1/D2 GenBank accession no. of closest species	Nucleotide substitutions to closest species	NO.of samples
		PYD96-12, PYD96-13, PYD96-17, PYD96-18, PYD96-19, PYD96-22, PCD96-2, PCD96-3, PYD98-3, PYD98-4, PYD98-6, PYD98-10, PYD98-12, PCD98-3, PYD100-1, PYD100-4, PYD100-7, PCD100-1, PCD100-2, PCD100-4			
Candida parapsilosis	77	PYD1-4, PYD1-8, PCD1-2, PCD1-4, PYD2-3, PCD2-1, PCD2-2, PCD2-3, PYD3-4, PYD3-7, PCD3-3, PYD5-8, PCD5-2, PCD5-4, PYD6-1, PYD6-2, PCD6-1, PCD6-2, PCD6-3, PYD88-2, PYD88-4, PYD88-5, PYD88-6, PYD88-9, PYD88-12, PCD88-1, PCD88-4, PCD88-5, PYD89-3, PYD89-5, PYD89-7, PCD89-1, PYD90-10, PYD90-14, PYD91-4, PYD91-8, PYD91-9, PCD91-2, PCD91-5, PYD92-1, PYD92-2, PYD92-3, PYD92-7, PYD92-9, PYD92-10, PCD92-1, PCD92-2, PCD92-4, PCD92-8, PCD92-9, PCD92-10, PCD95-11, PYD96-20, PYD96-24, PYD96-25, PCD96-1, PCD96-7, PCD96-8, PCD96-16, PYD98-2, PYD98-8, PYD98-9, PCD98-4, PCD98-6, PCD98-7, PCD98-9, PCD98-10, PYD99-1, PYD99-5, PYD99-10, PYD99-11, PCD99-3, PYD100-3, PYD100-5, PYD100-6, PYD100-9, PYD100-10	U45754	0-2	15
Candida tropicalis	32	PYD5-1, PYD5-2, PYD5-3, PYD5-4, PYD5-5, PYD5-7, PYD5-9, PCD5-1, PCD5-3, PYD90-15, PYD90-19, PCD90-2, PCD90-4, PCD90-6, PCD90-8, PCD90-10, PCD90-11, PCD90-19, PCD92-5, PYD94-3, PCD94-1, PCD94-5, PCD95-7, PCD95-8, PYD97-13, PCD97-1, PCD97-2,	NG054834	0-1	7

Taxa	No. of strain	strain	D1/D2 GenBank accession no. of closest species	Nucleotide substitutions to closest species	NO.of samples
		PCD97-3, PCD97-4, PCD97-7, PCD97-10, PYD98-11			
Lodderomyces elongisporus	2	PYD1-5, PCD91-1	U45763	0	2
Meyerozyma caribbica	2	PYD89-4, PCD97-8	AY187283	0-1	2
Schwanniomyces etchellsii	4	PYD2-1, PYD2-4, PYD96-4, PYD100-2	U45809	0	3
Metschnikowiaceae					
Candida vulturna	1	PCD91-4	JX515965	2	1
Phaffomycetaceae					
Cyberlindnera aff. *jadinii*	1	PYD99-2	EF550309	6	1
Cyberlindnera jadinii	6	PYD94-1, PYD94-4, PYD94-6, PCD94-2, PCD94-6, PYD96-11	EF550309	5	2
Pichiaceae					
Candida ethanolica	1	PYD90-25	U71073	0	1
Pichia kudriavzevii	10	PYD3-3, PYD90-6, PYD90-11, PYD90-24, PCD90-1, PCD90-7, PYD94-2, PYD94-5, PCD94-3, PCD94-4	EF550222	0-1	3
Pichia manshurica	5	PYD2-5, PYD90-8, PYD90-17, PYD90-21, PYD92-5	U75738	0-1	3
Saccharomycetaceae					
Candida glabrata	9	PYD89-2, PYD89-8, PCD90-20, PYD97-1, PYD97-7, PYD97-9, PYD97-11, PCD97-9, PCD97-11	U44808	1-2	3
Saccharomycetales incertae sedis					
Diutina rugosa	10	PCD3-4, PCD3-5, PCD3-6, PYD92-11, PYD92-12, PCD93-19, PYD95-18, PCD96-11, PCD96-13, PYD100-8	KT336717	0-2	6
Trichomonascaceae					
Wickerhamiella aff. *infanticola*	2	PYD90-26, PYD90-28	DQ438230	6	1

Taxa	No. of strain	strain	D1/D2 GenBank accession no. of closest species	Nucleotide substitutions to closest species	NO.of samples
Wickerhamiella pararugosa	8	PYD89-1, PCD90-17, PCD90-24, PYD92-8, PYD96-6, PYD97-4, PYD97-5, PYD97-6	U62306	0, 1, 2, 5	5
**Basidiomycota**					
**Agaricomycotina**					
Trichosporonaceae					
Trichosporon asahii	1	PYD90-27	AF105393	0	1
Trichosporon japonicum	6	PYD3-5, PYD88-1, PYD88-7, PYD88-8, PYD88-10, PYD95-8	AF308657	0	3
**Pucciniomycotina**					
Sporidiobolaceae					
Rhodotorula mucilaginosa	4	PYD3-6, PYD93-22, PYD98-5, PCD98-5	AF070432	0-1	3

Table S2: Identification of yeast from jejunal Pia samples based on the D1/D2 domain sequence analysis.

Taxa	No. of strain	strain	D1/D2 GenBank accession no. of closest species	Nucleotide substitutions to closest species	NO.of samples
**Ascomycota**					
**Pezizomycotina**					
Herpotrichiellaceae					
Exophiala dermatitidis	2	PYJ97-10, PYJ97-11	NG059225	0	1
Saccotheciaceae					
Aureobasidium elanogenum	2	PYJ95-5, PCJ95-4	FJ150926	1	1
**Saccharomycotina**					
Debaryomycetaceae					
Candida albicans	9	PYJ94-5, PCJ94-1, PCJ94-2, PCJ94-9, PYJ100-1, PYJ100-4, PYJ100-5, PYJ100-6, PYJ100-7	U45776	0-1	2
Candida metapsilosis	2	PCJ91-1, PYJ99-1	NG054815	0-1	2
Candida orthopsilosis	29	PCJ6-4, PYJ88-1, PYJ88-2, PYJ88-5, PYJ88-7, PCJ88-6, PCJ89-2, PCJ89-5, PCJ89-8, PCJ89-9, PYJ91-2, PYJ91-6, PYJ91-7, PCJ91-4, PCJ92-2, PYJ93-2, PYJ93-8, PYJ93-10, PYJ93-11, PYJ93-14, PYJ93-17, PCJ93-2, PCJ93-6, PCJ95-8, PCJ96-4, PYJ98-3, PYJ98-5, PYJ98-13, PCJ98-3,	FJ746056	0-1	9
Candida parapsilosis	40	PYJ1-2, PCJ1-2, PCJ1-5, PCJ1-6, PCJ2-1, PCJ2-2, PCJ2-3, PYJ3-1, PYJ6-1, PYJ6-2, PCJ6-1, PCJ6-2, PCJ6-3, PCJ6-5, PYJ88-4, PYJ88-6, PYJ88-8, PCJ88-1, PCJ88-2, PCJ88-3, PCJ88-5, PCJ89-3, PCJ89-4, PYJ91-1, PYJ91-5, PCJ91-5, PCJ92-4, PCJ93-1, PYJ95-3, PCJ95-1, PCJ95-3, PYJ96-4,	U45754	0-1	14

Taxa	No. of strain	strain	D1/D2 GenBank accession no. of closest species	Nucleotide substitutions to closest species	NO.of samples
Candida tropicalis	44	PYJ96-5, PCJ96-1, PCJ96-2, PYJ98-9, PCJ98-5, PYJ99-2, PCJ100-1, PCJ100-3 PYJ5-1, PYJ5-2, PYJ5-3, PYJ5-5, PCJ5-1, PCJ5-2, PCJ5-3, PYJ90-5, PYJ90-14, PYJ90-16, PYJ90-18, PYJ90-24, PCJ90-4, PCJ90-5, PCJ90-6, PCJ90-12, PCJ90-13, PCJ90-17, PCJ90-18, PCJ90-22, PYJ92-1, PCJ92-1, PCJ92-3, PCJ93-3, PYJ94-3, PYJ94-10, PYJ95-1, PYJ95-6, PCJ95-2, PCJ95-6, PCJ95-7, PCJ97-1, PCJ97-4, PCJ97-6, PYJ98-2, PYJ98-10, PYJ98-12, PYJ98-19, PCJ98-4, PYJ100-2, PYJ100-3, PCJ100-2, PCJ100-5, PCJ100-8	NG054834	0-2	9
Schwanniomyces etchellsii	3	PYJ2-1, PYJ2-2, PYJ2-3	U45809	0	1
Yamadazyma olivae	1	PYJ88-3	FJ71543	0	1
Metschnikowiaceae					
Candida vulturna	1	PCJ91-2	JX515965	2	1
Phaffomycetaceae					
Cyberlindnera aff. *jadinii*	2	PCJ90-11, PCJ90-16	EF550309	6	1
Cyberlindnera jadinii	8	PYJ92-4, PYJ93-5, PCJ93-4, PCJ93-5, PYJ94-7, PYJ94-8, PCJ94-7, PYJ96-9	EF550309	3-5	4
Pichiaceae					
Pichia kudriavzevii	6	PYJ90-10, PYJ90-17, PCJ90-7, PCJ90-19, PYJ94-2, PYJ94-9	EF550222	0-1	2
Pichia manshurica	9	PYJ89-4, PYJ89-8, PYJ90-4, PYJ90-20, PYJ90-25, PCJ90-1, PYJ92-2, PYJ98-22, PYJ99-3	U75738	0, 2	5
Saccharomycetaceae					
Candida glabrata	12	PYJ89-2, PYJ89-3, PYJ89-6, PYJ89-9, PYJ90-7, PYJ97-2, PYJ97-5, PYJ97-7, PYJ97-8, PYJ97-9, PYJ97-12, PCJ97-3	U44808	0-2	3
Saccharomycetales incertae sedis					

Taxa	No. of strain	strain	D1/D2 GenBank accession no. of closest species	Nucleotide substitutions to closest species	NO.of samples
Diutina rugosa	7	PYJ93-7, PYJ93-16, PYJ94-1, PCJ94-4, PCJ94-6, PCJ94-10, PYJ95-4	KT336717	0	3
Trichomonascaceae					
Wickerhamiella pararugosa	21	PYJ89-1, PYJ90-3, PYJ90-26, PCJ90-2, PCJ90-9, PYJ93-6, PYJ93-18, PYJ93-19, PCJ93-7, PCJ93-8, PYJ96-7, PYJ96-10, PYJ97-1, PYJ97-4, PYJ97-6, PCJ97-2, PCJ97-7, PYJ98-17, PYJ98-21, PYJ99-6, PYJ99-10	U62306	0-2	7
**Basidiomycota**					
**Agaricomycotina**					
Trichosporonaceae					
Trichosporon japonicum	1	PCJ3-1	AF308657	0	1
**Pucciniomycotina**					
Sporidiobolaceae					
Rhodotorula mucilaginosa	1	PYJ96-6	AF070432	0-1	1

Table S3: Identification of yeast from ileal Pia samples based on the D1/D2 domain sequence analysis.

Taxa	No. of strain	strain	D1/D2 GenBank accession no. of closest species	Nucleotide substitutions to closest species	NO.of samples
**Ascomycota**					
**Pezizomycotina**					
Herpotrichiellaceae					
Exophiala dermatitidis	3	PYI95-20, PYI97-6, PYI97-7	NG059225	0	2
Saccotheciaceae					
Aureobasidium elanogenum	3	PYI95-11, PYI95-16, PCI95-6	FJ150926	1	1
**Saccharomycotina**					
Debaryomycetaceae					
Candida albicans	10	PYI94-1, PYI94-5, PYI94-10, PCI94-2, PCI94-5, PYI100-4, PYI100-8, PCI100-1, PCI100-2, PCI100-5	U45776	0-1	2
Candida metapsilosis	6	PYI88-1, PYI88-2, PYI93-2, PYI93-11, PYI95-1, PYI100-6	NG054815	0-1	4
Candida orthopsilosis	45	PCI6-2, PYI89-3, PYI89-5, PYI89-6, PCI89-5, PCI89-7, PCI89-12, PCI89-13, PYI90-4, PYI91-2, PYI91-4, PYI93-1, PYI93-6, PYI93-10, PYI93-13, PCI93-1, PCI93-4, PCI93-8, PCI93-10, PYI95-2, PYI95-3, PYI95-7, PYI95-8, PYI95-10, PYI95-12, PYI95-13, PYI95-18, PCI95-1, PCI95-2, PCI95-7, PCI95-8, PCI95-9, PCI95-11, PCI95-13, PCI95-15, PCI95-18, PYI96-3, PYI96-4, PYI96-22, PCI96-1, PCI98-6, PYI99-5, PYI100-5, PYI100-10, PCI100-3	FJ746056	0-1	10
Candida parapsilosis	29	PYI1-1, PCI1-1, PCI1-7, PCI2-3, PCI2-5, PYI6-2, PCI88-3, PCI88-8, PCI89-6, PCI89-8, PCI89-9,	U45754	0-1	11

Taxa	No. of strain	strain	D1/D2 GenBank accession no. of closest species	Nucleotide substitutions to closest species	NO.of samples
Candida tropicalis	44	PYI91-7, PCI91-5, PCI91-17, PYI93-7, PYI93-8, PYI93-16, PCI93-2, PCI93-5, PCI93-9, PYI95-15, PCI95-10, PCI95-14, PYI96-13, PCI96-15, PCI98-2, PYI100-1, PCI100-4, PCI100-9 PYI3-6, PCI3-3, PYI5-1, PYI5-2, PYI5-3, PYI5-4, PYI5-7, PYI5-8, PCI5-1, PCI5-2, PCI5-3, PYI88-3, PYI88-4, PCI88-1, PCI88-2, PCI88-5, PCI88-7, PYI90-7, PYI90-9, PYI90-16, PCI90-1, PCI90-5, PCI90-7, PCI90-9, PCI90-11, PYI91-5, PYI91-6, PCI91-1, PCI91-6, PCI91-7, PCI91-10, PCI91-14, PYI92-1, PYI92-2, PYI92-6, PYI94-7, PYI94-8, PCI94-3, PCI94-6, PYI95-17, PYI98-6, PCI98-3, PYI99-8, PCI100-6	NG054834	0-1	11
Lodderomyces elongisporus			U45763	0	0
Meyerozyma caribbica	17	PYI96-1, PYI96-2, PYI96-7, PYI96-10, PYI96-14, PYI96-15, PYI96-20, PCI96-2, PCI96-3, PCI96-4, PCI96-5, PCI96-7, PCI96-8, PCI96-12, PCI96-13, PCI96-14, PCI96-17	AY187283	0-1	1
Schwanniomyces etchellsii	2	PYI2-2, PYI96-12	U45809	0	2
Yamadazyma olivae			FJ71543	0	0
Metschnikowiaceae					
Candida vulturna	1	PYI100-3	JX515965	2	1
Kodamaea ohmeri	1	PCI90-8	U45702	0	1
Phaffomycetaceae					
Cyberlindnera aff. *jadinii*	1	PYI91-12	EF550309	6	1
Cyberlindnera jadinii	6	PYI92-3, PYI92-4, PCI93-7, PCI94-1, PCI94-4, PYI97-9	EF550309	5	4
Pichiaceae					

Taxa	No. of strain	strain	D1/D2 GenBank accession no. of closest species	Nucleotide substitutions to closest species	NO.of samples
Pichia kudriavzevii	19	PYI3-1, PYI3-2, PYI3-3, PYI3-4, PYI3-5, PCI3-1, PCI3-2, PCI3-4, PYI90-3, PCI90-2, PCI90-10, PYI91-10, PYI91-15, PCI91-4, PYI94-2, PYI94-3, PYI94-4, PYI94-6, PYI94-9	EF550222	0-2	4
Pichia manshurica	6	PYI90-5, PYI90-15, PYI91-11, PYI92-7, PYI93-15, PYI99-11	U75738	0-1	5
Saccharomycetaceae					
Candida glabrata	6	PYI89-2, PYI89-4, PYI90-14, PYI97-1, PYI97-3, PYI97-5	U44808	1-2	3
Saccharomycetales incertae sedis					
Diutina rugosa	3	PYI93-12, PCI95-17, PYI96-19	KT336717	0	3
Trichomonascaceae					
Wickerhamiella pararugosa	17	PYI90-2, PYI90-12, PCI91-18, PYI92-8, PYI93-3, PYI93-9, PCI96-18, PYI97-2, PYI97-4, PYI97-8, PYI97-10, PCI97-1, PCI97-2, PCI97-3, PYI98-8, PYI99-6, PYI99-9	U62306	1-2	8
**Basidiomycota**					
**Agaricomycotina**					
Trichosporonaceae					
Trichosporon asahii	1	PYI90-11	AF105393	0	1
Trichosporon japonicum	1	PYI93-5	AF308657	0	1
**Pucciniomycotina**					
Sporidiobolaceae					
Rhodotorula mucilaginosa	1	PYI95-9	AF070432	0	1

Table S4: Identification of yeast from duodenal epithelium samples based on the D1/D2 domain sequence analysis.

Taxa	No. of strain	strain	D1/D2 GenBank accession no. of closest species	Nucleotide substitutions to closest species	NO.of samples
**Ascomycota**					
**Pezizomycotina**					
Herpotrichiellaceae					
Exophiala dermatitidis	1	YTD97-8	NG059225	0	1
**Saccharomycotina**					
Debaryomycetaceae					
Candida metapsilosis	1	YTD3-2	NG054815	0	1
Candida orthopsilosis	37	CTD3-3, CTD3-4, YTD88-2, YTD88-4, CTD88-8, YTD89-3, YTD89-4, YTD89-7, YTD89-8, CTD89-1, CTD89-2, YTD92-3, YTD92-7, YTD92-8, YTD92-9, CTD92-3, YTD93-4, YTD93-7, YTD95-1, CTD95-1, YTD96-3, YTD96-4, YTD96-7, YTD96-8, CTD96-1, CTD96-4, YTD97-3, YTD97-7, YTD97-12, CTD97-3, CTD97-7, YTD99-1, YTD99-3, YTD99-5, YTD99-7, YTD100-2, CTD100-4	FJ746056	0, 1, 4	10
Candida parapsilosis	73	YTD2-2, YTD2-3, YTD2-4, CTD2-1, CTD2-2, YTD3-1, YTD3-5, CTD3-5, YTD4-1, YTD4-2, CTD4-1, CTD4-2, YTD5-1, YTD5-2, YTD5-3, YTD6-1, CTD6-1, CTD6-2, YTD88-3, YTD88-6, CTD88-1, CTD88-3, CTD88-6, CTD88-7, CTD88-9, CTD88-10, YTD89-5, YTD89-6, YTD90-2, YTD90-3, YTD90-5, YTD90-9, CTD90-4, CTD90-5, YTD91-1, YTD91-3, YTD91-4, YTD91-6, YTD91-7, YTD91-8, CTD91-1, CTD91-3, CTD91-5, CTD91-6, YTD92-1, YTD92-2, YTD92-5,	U45754	0	15

Taxa	No. of strain	strain	D1/D2 GenBank accession no. of closest species	Nucleotide substitutions to closest species	NO.of samples
		CTD92-4, CTD92-6, CTD93-4, CTD93-5, YTD97-1, YTD97-6, YTD97-11, CTD97-1, CTD97-4, CTD97-6, YTD98-1, YTD98-2, YTD98-3, YTD98-7, YTD98-9, YTD98-10, CTD98-1, CTD98-3, CTD98-4, CTD98-5, CTD98-6, YTD99-4, CTD99-1, CTD99-2, CTD99-3, CTD100-3			
Candida tropicalis	2	CTD92-2, CTD97-8	NG054834	0	2
Candida zeylanoides	4	YTD100-3, YTD100-5, YTD100-9, CTD100-1	NG060834	0	1
Debaryomyces hansenii	1	CTD100-2	NG042634	0	1
Meyerozyma caribbica	2	YTD93-5, YTD93-6	EU348786	0	1
Schwanniomyces etchellsii	3	YTD2-1, YTD96-6, YTD97-10	U45809	0	3
Metschnikowiaceae					
Candida duobushaemulonii	1	CTD88-11	AJ508564	0	1
Candida vulturna	1	YTD92-4	JX515965	2	1
Pichiaceae					
Pichia manshurica	2	YTD93-1, YTD93-9	U75738	0	1
Saccharomycetaceae					
Kluyveromyces marxianus	1	CTD92-5	U94924	0	1
Saccharomycetales incertae sedis					
Diutina rugosa	5	YTD92-10, CTD92-1, CTD93-1, CTD93-2, CTD93-6	KT336717	0-2	2
Trichomonascaceae					
Wickerhamiella aff. infanticola	2	CTD88-2, CTD100-5	DQ438230	7	2
Wickerhamiella pararugosa	5	CTD3-2, YTD6-2, YTD97-9, CTD99-4, CTD99-6	U62306	0-2	4
Yarrowia lipolytica	3	CTD96-3, YTD97-4, YTD97-5	AM268450	0	2
**Basidiomycota**					

Taxa	No. of strain	strain	D1/D2 GenBank accession no. of closest species	Nucleotide substitutions to closest species	NO.of samples
**Agaricomycotina**					
Trichosporonaceae					
Trichosporon asahii	1	YTD96-1	AF105393	0	1
Trichosporon japonicum	3	YTD90-7, CTD90-1, CTD90-3	AF308657	0	1
**Pucciniomycotina**					
Sporidiobolaceae					
Rhodotorula mucilaginosa	14	YTD3-4, YTD6-3, YTD88-1, CTD88-5, YTD90-4, YTD90-8, YTD91-9, YTD91-10, YTD96-5, YTD97-2, CTD97-5, YTD98-6, YTD100-7, YTD100-8	AF070432	0-1	9

Table S5: Identification of yeast from jejunal epithelium samples based on the D1/D2 domain sequence analysis.

Taxa	No. of strain	strain	D1/D2 GenBank accession no. of closest species	Nucleotide substitutions to closest species	NO.of samples
**Ascomycota**					
**Pezizomycotina**					
Herpotrichiellaceae					
Exophiala dermatitidis	1	YTJ96-11	NG059225	0	1
**Saccharomycotina**					
Debaryomycetaceae					
Candida metapsilosis	1	CTJ93-3	NG054815	0	1
Candida orthopsilosis	31	CTJ3-2, YTJ89-3, YTJ89-5, YTJ89-6, CTJ89-2, CTJ89-4, CTJ89-6, YTJ90-8, YTJ92-4, YTJ92-6, CTJ92-5, YTJ93-2, YTJ93-3, YTJ93-4, CTJ93-7, YTJ96-4, YTJ96-10, CTJ96-1, YTJ97-3, YTJ97-5, CTJ97-2, CTJ98-1, CTJ98-2, CTJ98-3, CTJ98-5, CTJ98-9, YTJ100-3, YTJ100-5, YTJ100-7, CTJ100-4, CTJ100-7	FJ746056	0-2	9
Candida parapsilosis	52	YTJ2-1, YTJ2-2, YTJ2-3, CTJ2-1, CTJ2-2, YTJ3-1, CTJ3-1, YTJ4-2, YTJ4-3, YTJ4-4, CTJ4-1, CTJ4-2, YTJ6-1, YTJ6-2, YTJ6-4, CTJ6-1, CTJ6-2, YTJ89-1, YTJ89-7, CTJ89-1, CTJ89-5, YTJ90-3, YTJ90-5, CTJ90-1, YTJ91-1, YTJ91-4, YTJ91-7, YTJ91-8, YTJ92-1, YTJ92-2, YTJ92-8, CTJ92-1, CTJ92-2, CTJ92-3, YTJ93-5, YTJ93-6, YTJ93-7, YTJ93-10, CTJ93-1, YTJ96-2, YTJ96-9, CTJ97-4, CTJ97-5, CTJ97-6, YTJ98-2, YTJ98-3, YTJ98-4, CTJ98-7, YTJ99-1, CTJ99-8, YTJ100-4, YTJ100-8	U45754	0-1	14
Candida tropicalis	3	CTJ90-3, CTJ92-6, CTJ97-3	NG054834	0	3

Taxa	No. of strain	strain	D1/D2 GenBank accession no. of closest species	Nucleotide substitutions to closest species	NO.of samples
Candida zeylanoides	2	YTJ99-3, YTJ99-8	NG060834	0	1
Debaryomyces hansenii	2	CTJ99-3, CTJ100-3	NG042634	0	2
Schwanniomyces etchellsii	2	CTJ96-2, CTJ97-1	U45809	0	2
Yamadazyma olivae	1	YTJ4-1	FJ715430	0	1
Metschnikowiaceae					
Candida duobushaemulonii	1	CTJ92-4	AJ508564	0	1
Candida vulturna	4	YTJ89-4, CTJ98-8, YTJ99-5, YTJ99-7	JX515965	2	3
Kodamaea ohmeri	2	YTJ98-1, CTJ99-1	U45702	0	2
Pichiaceae					
Pichia kudriavzevii	1	CTJ90-2	EF550222	1	1
Pichia manshurica	2	YTJ90-7, YTJ92-7	U75738	0	2
Saccharomycetaceae					
Kluyveromyces marxianus	1	YTJ92-5	U94924	0	1
Saccharomycetales incertae sedis					
Diutina rugosa	6	CTJ93-5, CTJ93-6, CTJ96-3, YTJ97-4, CTJ100-5, CTJ100-6	KT336717	0-1	4
Trichomonascaceae					
Wickerhamiella infanticola	1	YTJ96-5	NG058278	6	1
Wickerhamiella pararugosa	7	YTJ90-4, YTJ90-6, YTJ97-7, YTJ97-9, YTJ99-2, YTJ99-4, CTJ99-7	U62306	0-2	3
**Basidiomycota**					
**Agaricomycotina**					
Trichosporonaceae					
Trichosporon japonicum	2	YTJ6-3, CTJ100-2	AF308657	0	2
**Pucciniomycotina**					

**Taxa**	**No. of strain**	**strain**	**D1/D2 GenBank accession no. of closest species**	**Nucleotide substitutions to closest species**	**NO.of samples**
Sporidiobolaceae					
Rhodotorula mucilaginosa	17	YTJ89-9, YTJ90-1, YTJ90-2, YTJ91-3, YTJ91-5, YTJ91-6, CTJ91-1, CTJ91-2, CTJ91-3, CTJ91-4, YTJ93-8, CTJ93-4, YTJ96-6, CTJ96-5, YTJ97-6, YTJ99-9, CTJ99-5	AF070432	0-1	7

Table S6: Identification of yeast from ileal epithelium samples based on the D1/D2 domain sequence analysis.

Taxa	No. of strain	strain	D1/D2 GenBank accession no. of closest species	Nucleotide substitutions to closest species	NO.of samples
**Ascomycota**					
**Saccharomycotina**					
Debaryomycetaceae					
Candida albicans	1	YTI100-6	U45776	1	1
Candida orthopsilosis	30	YTI1-1, YTI3-3, YTI88-2, CTI88-4, YTI90-1, YTI93-1, YTI93-2, CTI93-1, CTI93-6, YTI96-1, YTI96-3, YTI96-5, YTI96-6, YTI97-1, YTI97-3, CTI97-1, CTI97-2, CTI97-3, CTI97-4, YTI98-3, YTI98-4, YTI98-6, CTI98-4, CTI98-5, CTI98-7, YTI99-1, CTI99-5, YTI100-3, YTI100-10, CTI100-3	FJ746056	0, 1, 3, 5	10
Candida parapsilosis	57	YTI1-2, YTI1-3, YTI1-4, CTI1-1, CTI1-2, YTI2-1, YTI2-2, YTI2-3, YTI2-4, CTI2-1, CTI2-2, YTI3-1, YTI3-2, CTI3-1, CTI3-2, YTI4-1, YTI4-2, YTI4-3, CTI4-1, CTI4-2, YTI5-1, YTI88-1, YTI88-5, YTI88-6, CTI88-2, CTI88-3, CTI88-5, CTI88-6, YTI89-1, YTI90-3, YTI90-4, YTI90-5, CTI90-1, CTI90-2, CTI90-3, CTI90-4, CTI90-5, CTI92-1, CTI92-2, YTI93-5, YTI93-6, CTI93-4, CTI93-5, CTI96-3, YTI97-4, YTI97-5, CTI98-2, YTI99-2, YTI99-4, YTI99-5, YTI99-6, CTI99-1, CTI99-2, CTI99-6, YTI100-7, CTI100-1, CTI100-5	U45754	0-1	15
Candida tropicalis	1	YTI92-1	NG054834	0	1
Candida zeylanoides	1	YTI88-4	NG060834	2	1
Meyerozyma caribbica	2	YTI98-8, YTI98-9	EU348786	0	1
Schwanniomyces etchellsii	1	CTI98-3	U45809	0	1

Taxa	No. of strain	strain	D1/D2 GenBank accession no. of closest species	Nucleotide substitutions to closest species	NO.of samples
Metschnikowiaceae					
Candida duobushaemulonii	3	YTI100-8, CTI100-2, CTI100-4	AJ508564	0	1
Clavispora lusitaniae	1	YTI100-4	AY190538	0	1
Pichiaceae					
Pichia manshurica	5	YTI92-2, YTI92-3, YTI92-5, YTI92-6, CTI92-5	U75738	0	1
Saccharomycetaceae					
Kluyveromyces marxianus	2	CTI92-3, CTI92-4	U94924	0	1
Saccharomycetales incertae sedis					
Diutina rugosa	2	CTI96-4, YTI97-2	KT336717	0	2
Trichomonascaceae					
Wickerhamiella pararugosa	1	YTI98-1	U62306	0	1
**Basidiomycota**					
**Agaricomycotina**					
Trichosporonaceae					
Trichosporon asahii	1	YTI100-5	AF105393	0	1
**Pucciniomycotina**					
Agaricostilbaceae					
Sterigmatomyces elviae	1	YTI100-12	AF177415	0	1
Sporidiobolaceae					
Rhodotorula mucilaginosa	15	YTI3-4, YTI4-4, YTI5-2, YTI90-6, YTI90-7, YTI90-8, CTI90-6, YTI96-4, CTI96-5, CTI96-6, YTI97-8, CTI98-8, YTI99-7, YTI100-9, YTI100-11	AF070432	0-1	9
